# Verbal fluency and intrinsic brain activity in Alzheimer’s disease

**DOI:** 10.3325/cmj.2015.56.573

**Published:** 2015-12

**Authors:** Vanja Kljajević

**Affiliations:** University of the Basque Country, Vitoria & IKERBASQUE, Basque Foundation for Science, Bilbao, Spain *vanja.kljajevic@gmail.com*

## Why this topic?

Growing evidence from task-based functional magnetic resonance imaging (fMRI) studies consistently indicates network abnormalities in Alzheimer’s disease (AD) patients (1,2). While a picture is emerging on how these changes affect cognition and behavior at various stages of the disease, there is less understanding of the changes in functional connectivity between spatially distant brain areas in the absence of a specific task, ie, in a “resting state.” Resting state fMRI focuses on spontaneous fluctuations in the blood oxygen level dependent (BOLD) signal in distant yet temporally synchronous brain areas, which presumably reflect neural connectivity (3-5). It has been hypothesized that low-frequency (0.1-0.01 Hz) fluctuations in the BOLD signal may be attributed not only to spontaneous neuronal activity detectable in the absence of cognitive task, but also to specific functional domains. In other words, these temporally correlated fluctuations can indentify brain networks (6). The existing evidence suggests alterations in several resting-state networks in AD, such as the default mode network (7-9), fronto-parietal network, executive network, salience network (10), and language network (11).

Resting state functional connectivity (RS FC) changes in the AD brain involve both decreased and increased connectivity within specific networks. Interpretation of such alterations is far from trivial, and it remains largely unknown how they relate to continuing cognitive decline as the disease advances. Decreased RS FC presumably indicates lesion-driven disconnection processes, whereas increased connectivity indicates compensatory and adaptive connectivity mechanisms (11,12). While it remains unclear how such mechanisms allow relative preservation of cognitive function in the context of disease-related changes, the disease progression will eventually turn these mechanisms ineffective (9). In addition to changes in RS FC (13), early stages of AD are associated with pronounced hypometabolism in temporo-parietal regions in asymptomatic, ie, preclinical stage, as well as with atrophy originating in medial and lateral temporal areas and to some extent overlapping with hypometabolism in prodromal AD (14). Thus, as the disease develops, it affects more brain areas, and consequently cognitive changes become manifest in domains other than memory, including language. Anatomical changes in the AD brain and its structural connectivity have been reported in numerous post-mortem studies as well as in *in vivo* diffusion tensor imaging studies, while functional connectivity changes have been observed using other methods, such as rs- and task-based fMRI and positron emission tomography (PET).

Rs-fMRI is an excellent tool for assessing functional connectivity of distant brain areas in AD, because it does not require task performance in the scanner, which may be overwhelming to patients. In addition, the length of scanning in collecting rs-fMRI data are relatively short compared to task-based scanning sessions. The standard scanning duration in collecting rs-fMRI data are 5-7 min, although recent evidence suggests that scanning sessions that take 9-13 min result in improved reliability (15). Despite these obvious advantages, one reason for not utilizing this method to its full potential in clinical settings so far is related to some uncertainty regarding the relevance of intrinsic brain activity to behavior. However, the brain’s basal activity appears to be more relevant to the brain’s task-evoked activity than generally assumed (16). Recent studies suggest that FC of the RS brain networks reflects the connectivity of task-activated networks (17) and that its changes in disease may indicate residual abilities of the affected networks (18). Crucially, changes in RS FC of certain brain areas in AD significantly correlate with the patients’ poor performance on certain cognitive tests (19). For instance, abnormal RS FC between the left thalamus and left frontal areas (inferior frontal and middle frontal gyri) has been associated with AD patients’ immediate/delayed scores on Auditory Verbal Learning Test (19). In patients with mild cognitive impairment (MCI), who convert to AD at a rate of 10%-15% per year (6), RS FC values between the thalamus and temporo-parietal regions correlate with the patients’ scores on Mini Mental State Examination (13). Taken together, these studies indicate the relevance of RS thalamic connectivity with frontal and temporo-parietal brain areas to a range of cognitive functions that deteriorate across the stages of AD, such as memory and executive functions (speed of processing, working memory, attention). Furthermore, cognitively normal persons with increased levels of cortical β-amyloid also show abnormalities in RS FC in AD susceptible areas (6), including the thalamus (20), despite the absence of any cognitive symptoms.

It appears then that intrinsic brain activity assessed by rs-fMRI contains important information on the changes in AD brain that can be traced from preclinical and prodromal stages to dementia. Since aberrant patterns of RS FC in prodromal and dementia stages of AD have been associated with specific cognitive symptoms, as discussed above, these associations can be used in clinical settings to support diagnosis, monitoring of disease progression, and assessment of efficiency of disease-modifying treatments. The rest of the article discusses the relevance of thalamic RS FC with frontal areas for general verbal fluency in AD.

## Verbal fluency deficit in Alzheimer’s disease

AD patients regularly perform considerably worse on verbal fluency tests than cognitively healthy elderly subjects (21). In these tests, the task is to produce as many words as possible in 1 minute, provided that the produced words belong to a specific category (category test) or begin with a specific letter (letter test). Impaired performance on verbal fluency tests in AD has been associated with rapid disease progression, probably reflecting advanced deterioration of the frontal networks (22).

Verbal fluency tests are often considered the tests of executive function, but as simple lexical tasks – cued word generation – they also implicate language. Even though some evidence suggests that category fluency better reflects verbal abilities whereas letter fluency better reflects executive function, not all findings support such a distinction (23). It has been long recognized that it may not be possible to disentangle the language system from the executive system (24), in particular in tasks that require a rapid turn of cognitive operations, as is the case in cued word retrieval. Tests of verbal fluency trigger a set of processes such as defining a search strategy, performing search either by a semantic category cue or letter cue, retrieving words from the semantic or phonological memory, assessing their semantic/ phonological representations, inhibiting words that do not match the search criteria, articulating the retrieved word, keeping track of the retrieved words while continuing the search, focusing on the task while keeping track of time given the time limit, and so on (25). Thus, although verbal fluency tests require both executive functioning and verbal abilities, they are not “pure” tests of either. Nevertheless, these tests remain a critical tool for diagnosis and monitoring of changes in cognitive status in a range of conditions.

Attempts to determine the neural basis of verbal fluency indicate a special role of Broca’s area in these tasks, while also implicating a number of other areas. An early task-based fMRI study of covert cued word generation in healthy subjects that used both letter and category fluency tests (26) reported involvement of the left inferior frontal gyrus (LIFG), superior temporal gyrus, posterior superior temporal-inferior parietal areas, insula, retrosplenial area, as well as pulvinar and dorsomedial thalamic regions. Interestingly, this study also reported a functional segregation of Broca’s area for fluency tests, with Brodmann area (BA) 45 supporting category fluency and BA 44/6 supporting letter fluency. The role of BA 45 in category fluency and BA 44 in letter fluency has been confirmed in other task-based fMRI studies among healthy participants (27) and aphasic patients (28). These findings have been interpreted as indicating that BA 45 may contribute to the word retrieval through their meaning, and BA 44/6 may contribute to word retrieval via an articulatory code (26).

## Broca’s area-thalamus: functional connectivity at rest

A seminal study on RS FC of language networks that involved 970 healthy participants scanned at 22 institutions across the world suggests that the brain at rest reveals an extended language network, implicating the classical language areas (Broca’s, Wernicke’s) as well as a range of prefrontal, temporal, and parietal regions along with subcortical structures (29). Other studies confirmed the reliability and stability of the language network at rest (30). Thus, recent findings on low frequency fluctuations in FC underlying language networks (12,17,29,31) on the one hand, and findings from task-based fMRI studies indicating involvement of Broca’s region in verbal fluency on the other support an association between AD patients’ verbal fluency decline and RS FC abnormalities involving this region. An intriguing possibility would be that functional segregation of Broca’s area into a region responsible for category and a region responsible for letter fluency (26) would be reflected in the connectivity patterns of the AD brain at rest.

In addition, the thalamus appears to be implicated in a range of language-related tasks, eg, word generation, speech production, picture naming, reading, and syntactic and semantic processing (28,32-35). The thalamus is presumed to play a neuroregulatory role in language, by recruiting controlled processes when relying on automatic processes is not sufficient (32), or the role of a “central monitor” for cortical language-related activations (33). Given the thalamic role in control processes associated with lexical retrieval and thalamic changes in the AD brain, changes in functional interconnectivity between Broca’s area and thalamic regions at rest are likely to be related to verbal fluency decline in AD.

Early evidence for the role of the left pulvinar and some sections of the left ventrolateral nucleus in word retrieval comes from electrostimulation studies with patients undergoing awake brain surgery for the treatment of dyskynesias (36,37). An early model of the thalamic role in language postulated connections between the pulvinar and temporoparietal cortical areas, and between the left dorsomedial nucleus and frontal cortical language areas (38). A recent diffusion tensor imaging study involving 10 healthy young subjects demonstrated direct structural connectivity between the pulvinar and ventral anterior nucleus with Broca’s area (39). While evidence indicates that these thalamic regions are implicated in semantics (40), or in integration of semantic and phonological information (39), the dorsomedial nucleus may be contributing to phonological word retrieval. This nucleus has rich interconnectivity with prefrontal areas (41) and it plays a critical role in executive functions (42) and language (43,44). Shrinkage of the left dorsomedial thalamic nucleus correlates with the decline of executive function and general cognitive decline in AD patients (45). The role of this nucleus in working memory has been confirmed in studies using different research methods (42). Furthermore, damage to the left dorsomedial thalamic nucleus has been associated with word finding difficulty and verbal working memory deficits (phonological loop) in thalamic aphasia (46). Finally, RS FC of the dorsomedial thalamic nucleus with prefrontal areas has been established (47), and decreased FC at rest between the thalamus and left middle (although not inferior) frontal gyrus was found in MCI (13). Thus, it is likely that this nucleus, in addition to the pulvinar, contributes to the dysfunction of networks associated with verbal fluency decline in AD. Moreover, if letter fluency better reflects executive functions, as sometimes claimed (48), then AD patients’ letter fluency scores may correlate with FC values between this nucleus and BA 44. Future studies will test these hypotheses and investigate how RS functional connectivity associated with word deficits in patients on the AD spectrum (49) differs from the RS FC associated with word deficits in primary progressive aphasia with thalamic atrophy and in post-stroke thalamic aphasia due to focal lesions (eg, tuberothalamic infarction) (50).

Since considerable atrophic changes in frontal areas do not appear in early stages of AD (14), whereas regional thalamic atrophy is detectable in prodromal stage (13), abnormalities in RS FC between Broca’s area and thalamic regions are likely to appear due to thalamic dysfunction. Decreased RS FC of the thalamus was found in cognitively normal persons with increased cortical β-amyloid load (20), ie, in preclinical AD, where cognitive symptoms such as verbal fluency decline are not present. Thus, abnormal RS FC between Broca’s area and thalamic regions that correlates with verbal fluency decline in AD patients is potentially a useful indicator of transition toward advanced stages of the disease (prodromal to mild-to-moderate). In addition, RS FC between these areas and their association with verbal fluency may be useful in the evaluation of treatments that intend to slow down the disease progression.

In conclusion, it is important to better integrate cognitive neuropsychological and rs-fMRI data in clinical settings. This may provide a better insight into transition toward advanced stages of AD and help in the evaluation of treatments intended to slow down progression of the disease. Such integration of data are expected, given the increasing role of neuroimaging assessment in diagnosis and monitoring of AD, and the relative ease of incorporating rs-fMRI scanning into general scanning protocols.
